# Development and Validation of an LC-MS/MS-Based Screening Method for Determination of Salbutamol in Urine for Doping Control Using a Surrogate Analyte Approach with Stable Isotope-Labeled Salbutamol (Salbutamol-d9)

**DOI:** 10.5812/ijpr-169531

**Published:** 2026-08-19

**Authors:** Zeinab Srour, Farzad Kobarfard, Zahra Sheikholislam, Mehrdad Faizi, Bahram Daraei

**Affiliations:** 1 Department of Pharmacology and Toxicology, School of Pharmacy, Shahid Beheshti University of Medical Sciences, Tehran, Iran; 2 Department of Medicinal Chemistry, School of Pharmacy, Shahid Beheshti University of Medical Sciences, Tehran, Iran; 3 Department of Toxicology and Pharmacology, School of Pharmacy, Shahid Beheshti University of Medical Sciences, Tehran, Iran

**Keywords:** Doping Control, Labeled Analyte, LC-MS/MS, Salbutamol, Surrogate Analyte

## Abstract

**Background:**

Athletic doping has long received attention in the sports community. Salbutamol is prohibited by the World Anti-Doping Agency (WADA) both in and out of competition, with a urinary concentration threshold of 1000 ng/mL defining the legal limit. Traditional liquid chromatography-tandem mass spectrometry (LC-MS/MS) analytical methods that use an internal standard cannot fully overcome matrix effects and biological variation, requiring revalidation for different biological samples; this process can be time-consuming and can remain prone to matrix-related errors.

**Objectives:**

To develop a novel approach for screening salbutamol in urine that overcomes the limitations of traditional LC-MS/MS methods and improves analytical reliability and efficiency across biological matrices.

**Methods:**

A surrogate analyte-based LC-MS/MS method was developed for the quantitative determination of salbutamol in human urine, using salbutamol-d9 as a stable isotope-labeled surrogate analyte. During method optimization, concentrations ranging from 1 ppm to 0.1 ppb were analyzed in aqueous and urine samples to evaluate extraction efficiency, purification, and matrix effects. Molar-equivalent concentrations of unlabeled salbutamol (1000 ng/mL, the WADA legal limit) and labeled salbutamol (1039 ng/mL) were analyzed in both matrices to validate the surrogate analyte approach and confirm comparable peak areas. To evaluate the applicability of the method, urine samples were collected from healthy volunteers after oral administration of 4 mg salbutamol. All samples were extracted under alkaline conditions using liquid–liquid extraction and analyzed by LC-MS/MS in positive-ion mode with multiple reaction monitoring (MRM). The labeled compound served as a reference for interpretation of the results. Method validation was performed according to international guidelines, including assessments of linearity, precision, accuracy, and sensitivity.

**Results:**

The method demonstrated excellent linearity (R^2^ > 0.99), with intra- and inter-day precision within 8% RSD and acceptable accuracy. The limits of detection (LOD) and quantification (LOQ) were 0.3 and 1 ng/mL, respectively. Analysis of urine from healthy volunteers following a 4 mg oral dose confirmed reliable quantification for up to 24 hours, demonstrating applicability to biological matrices.

**Conclusions:**

These findings indicate that this isotope-based surrogate analyte approach eliminates the need for calibration curves, compensates for matrix effects and sample preparation errors, and improves analytical reliability and efficiency across biological matrices.

## 1. Background

Over the years, doping has increasingly become a major challenge for the sports community, in parallel with the development and expansion of professional and amateur sports and improvements in athletes' social and economic status (1, 2). Despite substantial advances in education and prevention, combating doping remains a key priority in sports policies (3). In 1999, the World Anti-Doping Agency (WADA) was established to promote prevention, detection, and sanctioning as core components of anti-doping policies. Given the high volume of samples and the number of doping substances, particularly during major events such as the Olympics, monitoring prohibited substances should be rapid and precise and capable of detecting concentrations above or below the thresholds set by WADA (4).

Salbutamol, a beta-2 adrenergic receptor agonist, is commonly used to treat asthma and chronic obstructive pulmonary disease (COPD). Except for inhaled salbutamol, systemic use is prohibited because of its potential anabolic effects and performance-enhancing benefits (5, 6). To ensure compliance with established thresholds, urinary salbutamol concentrations are routinely monitored, and concentrations exceeding 1000 ng/mL are considered a doping violation by WADA (7, 8). A urinary salbutamol concentration above 1000 ng/mL indicates systemic use (oral or injectable), whereas lower levels suggest inhaled use. However, interindividual pharmacokinetic variability, including differences in absorption, distribution, metabolism, and excretion, makes dosage estimation from urinary levels challenging (9).

In Iran, as in many countries, doping remains a major challenge in sports, particularly because of the widespread misuse of anabolic steroids and other prohibited substances. Despite efforts to educate athletes, misconceptions about the legality of certain drugs and supplements persist, underscoring the need for accurate, efficient, and accessible screening methods to monitor and prevent doping practices (10).

Various analytical methods, including enzyme-linked immunosorbent assay (ELISA), immunoassays, gas chromatography (GC), and liquid chromatography (LC), have been used to quantify salbutamol (11-13). Although gas chromatography-mass spectrometry (GC-MS) and GC-MS/MS methods have demonstrated reliability, they often require time-consuming derivatization steps. In contrast, LC-MS and LC-MS/MS techniques have emerged as efficient and cost-effective alternatives by eliminating the need for derivatization and offering improved sensitivity and precision, particularly when combined with solid-phase extraction (SPE) (14-16). Classical LC-MS methods for analyzing salbutamol in urine face challenges due to matrix effects, which vary considerably among individual samples and across biological matrices such as blood and saliva. These effects, which often cause ion suppression, can compromise analytical accuracy. To address these issues, internal standards are commonly used. These compounds, which are absent from unknown samples, are added in fixed amounts and processed alongside the analyte. By maintaining a consistent signal ratio, internal standards effectively compensate for analyte losses during sample preparation (17-19).

In traditional methods, calibration curves are generated by adding known concentrations of the analyte to samples containing a fixed internal standard. This approach provides reliable quantification by correcting for analyte loss. However, isotopically labeled internal standards have become the preferred choice for LC-MS/MS analyses because they mimic the analyte's behavior and more effectively compensate for matrix-induced signal changes. Matrix variation presents an additional challenge and requires revalidation of methods for different biological samples. For instance, a method validated for urine cannot be directly applied to blood without modification. This process is time-consuming and can leave residual matrix-related errors, emphasizing the need for innovative solutions. To overcome these limitations, the present study developed a novel method using stable isotope-labeled compounds, known as surrogate analytes, to streamline salbutamol screening in urine. Salbutamol (molecular mass, 239.31 g/mol) and its labeled derivative (molecular mass, 248.37 g/mol) share similar physicochemical properties, such as solubility, and are similarly affected by matrix effects. When equimolar concentrations of these compounds are analyzed, they generate signals with comparable peak areas. In this method, labeled salbutamol is spiked into urine samples at a fixed concentration of 1039 ng/mL, which is molar-equivalent to 1000 ng/mL of unlabeled salbutamol, the threshold value used in this study. Upon LC-MS/MS analysis, 2 signals are generated: one for unlabeled salbutamol and another for labeled salbutamol. Although these compounds elute simultaneously because of their similar physicochemical properties, their different molecular masses allow clear differentiation in the mass spectrometer. By extracting these signals from the total ion chromatogram (TIC), 2 distinct peaks with specific peak areas are obtained. The peak area of labeled salbutamol, representing the fixed molar-equivalent concentration, serves as a reference for determining doping status. If the peak area of unlabeled salbutamol is smaller than that of labeled salbutamol, the sample is classified as non-doping. Conversely, if the peak area of unlabeled salbutamol exceeds that of labeled salbutamol, the sample is classified as doping. This approach eliminates the need to construct calibration curves and simplifies the analytical process.

This method compensates for errors during sample preparation and for matrix effects because both unlabeled and labeled salbutamol are affected similarly. Using a labeled compound at a fixed molar-equivalent concentration helps reduce matrix effects and improves accuracy without the need for revalidation for different biological samples. In addition, the time required for method development and validation is reduced, making this approach faster and more practical than traditional methods. Collectively, this method provides a robust, reliable, and efficient alternative for salbutamol analysis, streamlining the workflow and supporting accurate quantification in anti-doping testing.

## 2. Objectives

The objective of this study was to develop a surrogate analyte-based LC-MS/MS approach for salbutamol screening in urine that could compensate for matrix and sample-preparation effects and improve analytical reliability and efficiency across biological matrices.

## 3. Methods

The method was divided into three stages: analysis of concentrations ranging from 1 ppm to 0.1 ppb in aqueous solutions, analysis of the same concentration range in urine samples, and analysis of urine samples from individuals following oral administration of salbutamol.

### 3.1. Chemicals and Reagents

Salbutamol (molecular weight, 239 g mol^-1^; purity > 99%; ChemCruz) and its labeled derivative, salbutamol-d9 (molecular weight, 248.37 g mol^-1^; purity > 99%; Cayman Chemical Company), were used. High-performance liquid chromatography (HPLC)-grade water, ethanol, methanol, and acetonitrile were obtained from Merck (Darmstadt, Germany) and evaluated for solubility, stability, and blankness. Ethanol was selected as the solvent for dissolution. Additional reagents, including sodium bicarbonate and ethyl acetate, were of analytical grade and were used during sample preparation and extraction.

### 3.2. Accurate Calculation of Labeled Salbutamol Equivalent to the WADA Legal Threshold

Despite injection of equal mass amounts of unlabeled and labeled salbutamol, the resulting peaks did not yield equal peak areas. This difference may be attributable to several factors, including differences in molecular weight and analyte purity. In this case, both unlabeled and labeled analytes had identical purities (99.99%); therefore, purity did not contribute to the calculation.

Because of the difference in molecular weight between unlabeled (239 g/mol) and labeled (248.37 g/mol) salbutamol, equal mass amounts do not contain equal molar amounts. Consequently, the peak areas are not equivalent.

To obtain equal signal intensities (ie, comparable peak areas) for unlabeled and labeled salbutamol in LC-MS/MS analysis, their molar concentrations must be equal. This requires preparation of solutions with the same molar concentration of each compound.

To calculate the mass of labeled salbutamol that is molar-equivalent to 1000 ng of unlabeled salbutamol, the WADA-established legal limit used in this study, a molar-equivalency calculation was performed based on molecular weights (molar-mass correction). A proportion was established between the molecular weights of the unlabeled and labeled analytes and the mass of the unlabeled analyte:

$$W_{L}=W_{\mathrm{UL}}\frac{{\mathrm{MW}}_{L}}{{\mathrm{MW}}_{\mathrm{UL}}}$$

W_L_: weight of labeled analyte

W_UL_: weight of unlabeled analyte

MW_L_: molecular weight of labeled analyte

MW_UL_: molecular weight of unlabeled analyte

The calculation showed that 1039 ng of labeled salbutamol is molar-equivalent to 1000 ng of unlabeled salbutamol. When these molar-equivalent amounts were spiked into urine samples, followed by sample preparation and LC-MS/MS injection, the resulting chromatographic signals (peak areas) for the unlabeled and labeled analytes were equal. This equivalence was experimentally confirmed.

### 3.3. Standard Solutions

A standard ethanolic solution of unlabeled and labeled salbutamol (1 mg/mL) was prepared using the weight-to-weight method. Serial dilutions of the stock solution were prepared as working solutions for method development and validation. All solutions were stored at -20 °C.

### 3.4. Urine Sample Preparation

Three types of urine samples were prepared: Urine spiked with a salbutamol solution, urine spiked with a salbutamol-d9 solution, and urine spiked with a mixture containing varying concentrations of salbutamol and a fixed concentration of salbutamol-d9 equivalent to the legal threshold. The final concentration of labeled salbutamol was 1039 ng/mL, which is molar-equivalent to 1000 ng/mL of unlabeled salbutamol.

For the preparation and purification of urine samples, 0.2 mL of salbutamol solution, 0.2 mL of salbutamol-d9 solution, or 0.2 mL of their mixture was spiked into 0.2 mL of urine. A 0.2 mL aliquot of spiked urine was mixed with 0.2 mL of sodium bicarbonate (0.5 mol/L in deionized water) and 4 mL of ethyl acetate. The mixture was vortexed for 2 minutes using a vortex mixer (Dragon Lab Instrument, Beijing, China) and centrifuged at 16,000 × g for 15 minutes. The supernatant was carefully transferred to a microtube and evaporated to dryness under a nitrogen stream. The residue was reconstituted in 0.2 mL of an acetonitrile-water mixture (70:30, v/v) and subsequently analyzed using LC-MS/MS according to the U.S. Food and Drug Administration bioanalytical method-validation guidance cited in the submitted manuscript. Previously published studies have used different sample-preparation strategies, including liquid-liquid extraction with organic solvents, solid-phase extraction, or simple dilution followed by the addition of an internal standard. Compared with these approaches, the present method uses a simple liquid-liquid extraction procedure combined with a surrogate analyte strategy, providing effective matrix cleanup while maintaining high sensitivity and simplicity (20, 21).

### 3.5. LC-MS/MS Conditions

#### 3.5.1. Liquid Chromatography-Tandem Mass Spectrometry

Samples were analyzed using an Agilent 6410 ESI triple-quadrupole LC/MS system. The mass spectrometer was calibrated and optimized for the detection of salbutamol. For this purpose, a 1 ppm ethanolic solution of both unlabeled and labeled salbutamol was infused directly into the mass detector. Positive-ion mode was selected because salbutamol can form cations. Precursor ions were identified by full-scan analysis, followed by product-ion scanning to determine product ions using optimized collision energy (CE) and fragmentor voltage. Multiple reaction monitoring (MRM) was used to monitor precursor and product ions for selective detection.

Chromatographic separation was achieved using a reversed-phase InertSustain C18 HPLC column (125 mm; 3 μm particle size; GL Sciences). Methanol/water (80/20) was used as the mobile phase. The flow rate and injection volume were 0.8 mL/min and 10 μL, respectively. Each sample had a total LC-MS/MS run time of approximately 2 minutes. Mass data were analyzed using Data Acquisition and Quantitative Analysis software.

During method optimization, all studied concentrations (1 ppm to 0.1 ppb) were initially analyzed in aqueous solutions. Subsequently, to evaluate the extraction and purification processes and assess the effect of the urine matrix, the same concentrations were analyzed in urine samples. To validate the surrogate analyte method and confirm that injection of molar-equivalent amounts of unlabeled and labeled salbutamol resulted in comparable peak areas, a mixture containing 1000 ng/mL unlabeled salbutamol and 1039 ng/mL labeled salbutamol was prepared in aqueous solution and urine samples and analyzed using LC-MS/MS.

### 3.6. Method Validation

The method was validated in accordance with established guidelines to ensure reliability and reproducibility. Validation parameters included linearity, precision, accuracy, and sensitivity. Calibration curves were generated over a range of 125 to 2000 ppb unlabeled salbutamol with a fixed concentration of 1039 ng/mL salbutamol-d9, which was molar-equivalent to the 1000 ng/mL legal threshold. Correlation coefficients (R^2^) were used to assess linearity. Precision was evaluated by determining intra-day and inter-day relative standard deviations (RSDs) through repeated sample analysis. Three replicate analyses were performed on a single day, followed by analyses over 3 different days to assess reproducibility and consistency. Accuracy was assessed by calculating the relative error (RE) of measured concentrations. Sensitivity was determined by calculating LOD and LOQ, defined as signal-to-noise ratios (S/N) ≥ 3 and ≥ 10, respectively.

### 3.7. Application to Human Samples

Urine samples were collected from healthy volunteers after the administration of a single oral dose of 4 mg salbutamol. Samples were analyzed at 3, 24, 48, and 72 hours after administration to evaluate method applicability and the urinary excretion profile of salbutamol. Samples were collected under controlled conditions and in accordance with a clinical protocol approved by the Iran National Committee for Ethics in Biological Research. The samples were stored at -20 °C until analysis. The study was approved by the Research Ethics Committee of the School of Pharmacy and Nursing and Midwifery, Shahid Beheshti University of Medical Sciences (code: IR.SBMU.PHARMACY.REC.1403.267).

The normal refractive index range for urine is approximately 1.336 to 1.349. Values outside this range may indicate issues such as overhydration or sample manipulation, although upper-limit values are rarely a concern in practice. This range was considered normal throughout the study.

### 3.8. Statistical Analysis

Statistical analyses were performed using Microsoft Excel. Data are expressed as the mean ± SD from n = 3 replicate measurements. Linearity was assessed by linear regression analysis using the least-squares method and reported as the coefficient of determination (R^2^). Intra-day and inter-day precision were expressed as relative standard deviation (%RSD) and calculated from repeated measurements within the same day and across different days, respectively. Accuracy was evaluated as percentage recovery by comparing measured concentrations with nominal (spiked) concentrations. The LOD and LOQ were estimated using signal-to-noise ratios of ≥ 3 and ≥ 10, respectively.

## 4. Results

**Figure 1.**
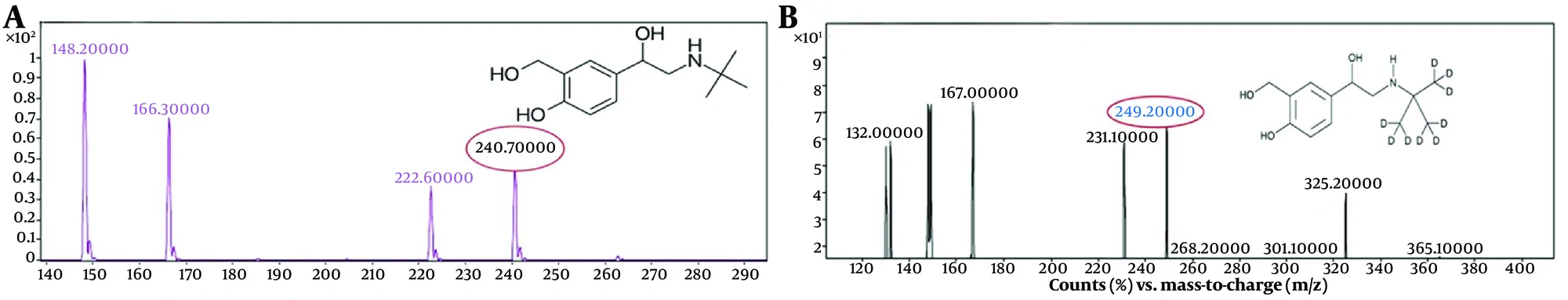
Mass spectra of unlabeled and labeled salbutamol. (A) Mass spectrum of unlabeled salbutamol (m/z 240.70). (B) Mass spectrum of labeled salbutamol (salbutamol-d9; m/z 249.20).

The development of analytical approaches for detecting doping agents is challenging because of the structural diversity of compounds on the WADA prohibited list and the need for high sensitivity and selectivity. The choice of analytical technique depends on the specific doping agents targeted and the desired balance among sensitivity, selectivity, and matrix effects (13, 14). Emerging techniques, such as LC-MS/MS-based surrogate-analyte approaches, demonstrate substantial potential for detecting various doping agents with high sensitivity and selectivity (13). In the present study, initial optimization of mass-spectrometer conditions was crucial for the selective detection of salbutamol and its labeled form. In full-scan mode (MS1), the parent ions of unlabeled salbutamol (m/z 240.70) and labeled salbutamol (m/z 249.20) were identified. Selecting the most abundant ion as the parent ion ensured high sensitivity and specificity. A mass range of ± 2 Da further enhanced the accuracy of ion selection, as shown in Figure 1.

**Figure 2.**
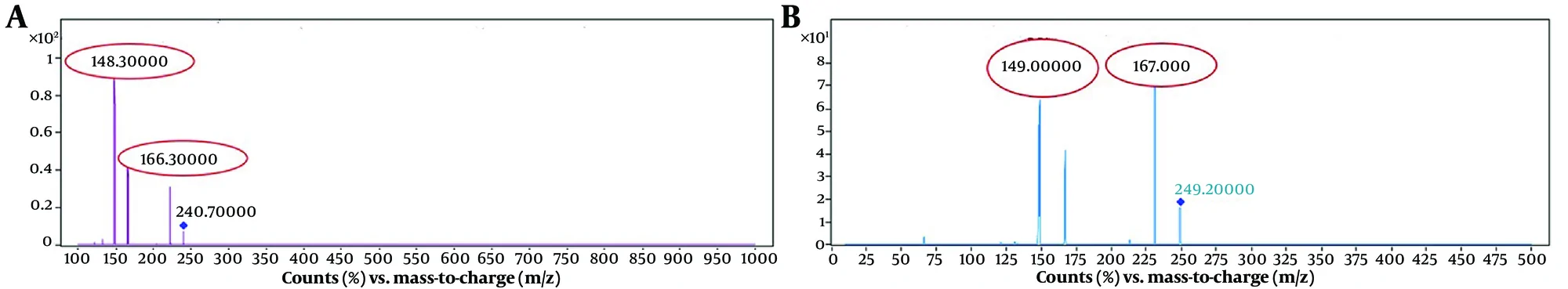
Mass spectra showing the precursor and product ions of unlabeled and labeled salbutamol. (A) Unlabeled salbutamol: precursor ion at m/z 240.7 and daughter ions at m/z 148.30 and m/z 166.30. (B) Labeled salbutamol (salbutamol-d9): precursor ion at m/z 249.20 and daughter ions at m/z 149.00 and m/z 167.00.

Product-ion scanning enabled identification of daughter ions resulting from fragmentation of the parent ions. For unlabeled salbutamol, daughter ions were observed at m/z 148 and m/z 166, whereas for salbutamol-d9, daughter ions were detected at m/z 149 and m/z 167. These results, shown in Figure 2, confirm successful fragmentation of both analytes under optimized CE and fragmentor settings. Differences in CE and fragmentor values between unlabeled and labeled salbutamol (10 and 10 for unlabeled; 20 and 40 for labeled) highlight the importance of tailoring optimization for each compound to achieve optimal fragmentation. Previous studies have demonstrated that optimizing fragmentation conditions is crucial for sensitive and informative mass-spectrometry results (16, 17), consistent with the use of different CE and fragmentor values for unlabeled and labeled salbutamol.

For simultaneous monitoring, an MRM system was set up for each ion. Fragmentor voltage, CE, and molecular masses of the parent and daughter ions were configured, as presented in Table 1. The dwell time for monitoring each ion during each cycle was set to 52 milliseconds. This short dwell time enables rapid cycling through multiple transitions, facilitating simultaneous monitoring of numerous compounds. This approach is widely used for the quantitative analysis of drugs, metabolites, and other small molecules in complex biological samples (22).

**Table 1.** Results of Ion Monitoring with MRM

Analyte	Mode of Ionization	Molecular Weight	Precursor Ion (m/z)	Product ion (m/z)	CE	Fragmentor
**Unlabeled salbutamol**	Positive	239.00	240	148 / 166	10	10
**Salbutamol-d9**	Positive	248.37	249	149 / 167	20	40

**Figure 3.**
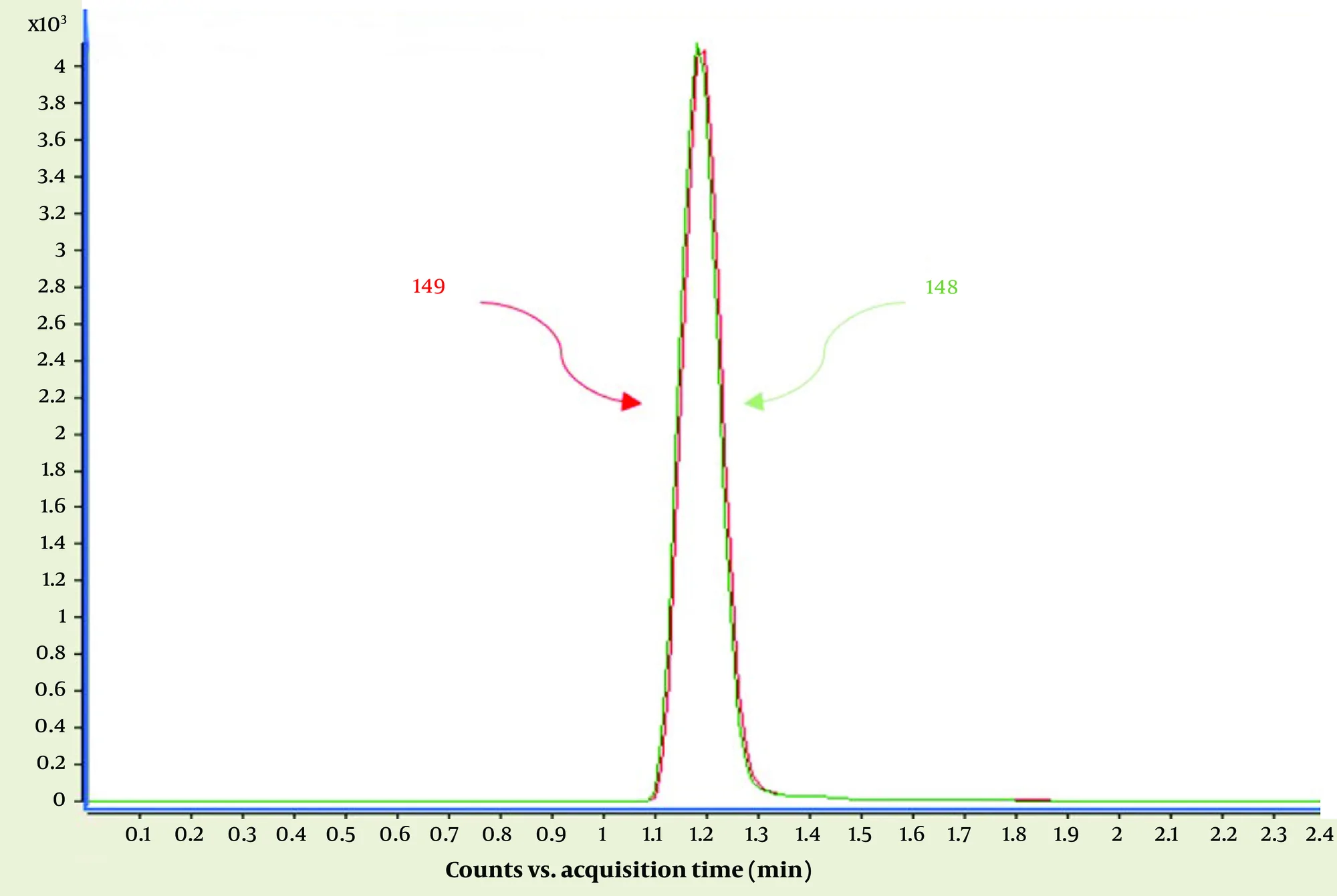
Chromatogram of the mixed aqueous sample. The green peak represents unlabeled salbutamol (m/z 240 → m/z 148), and the red peak represents isotopically labeled salbutamol (m/z 249 → m/z 149). Injection of molar-equivalent concentrations of unlabeled salbutamol (1000 ng/mL) and labeled salbutamol (1039 ng/mL) into the LC-MS/MS system resulted in comparable peak areas for both analytes.

**Figure 4.**
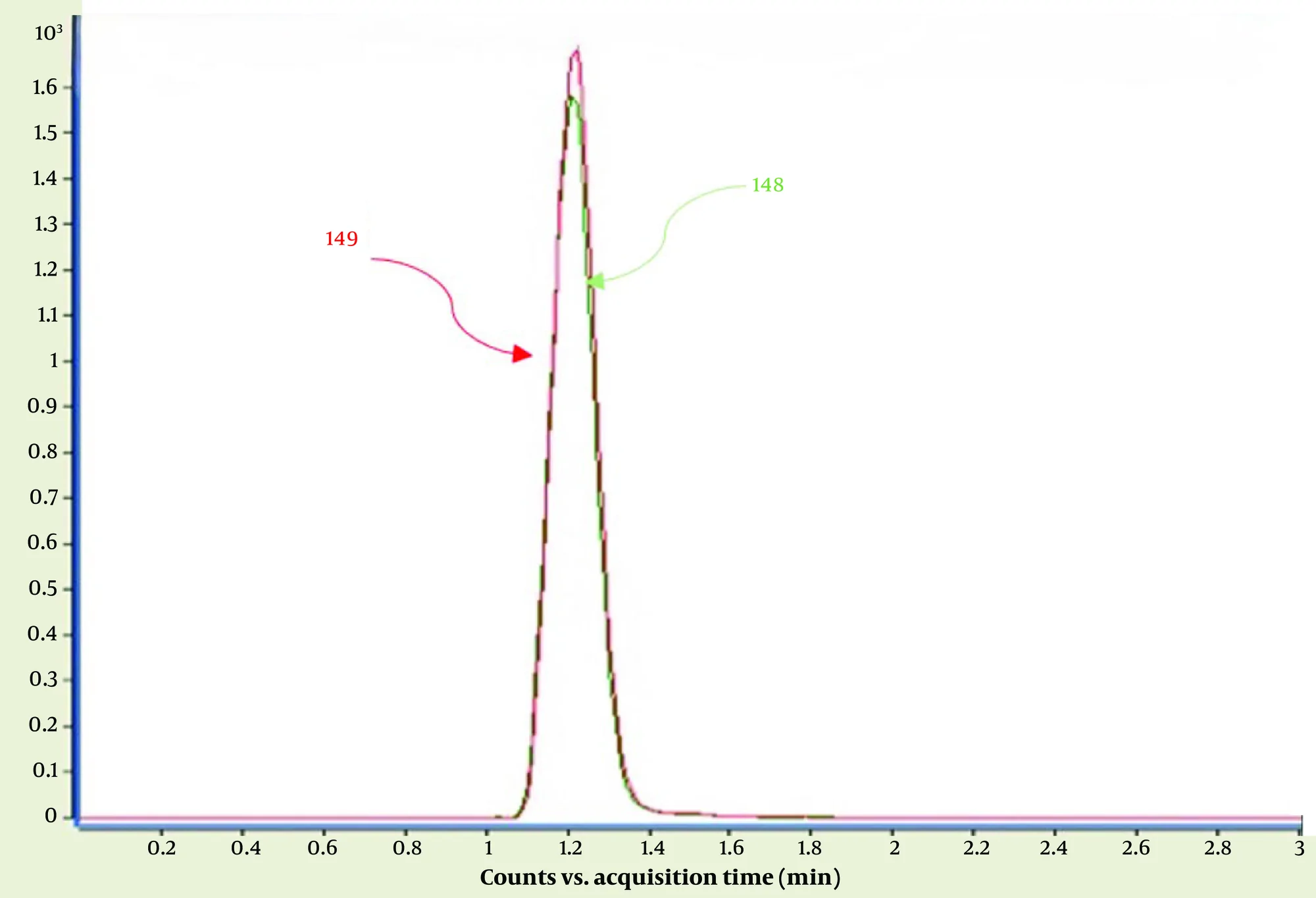
Chromatogram of the mixed urine sample. The green peak represents unlabeled salbutamol (m/z 240 → m/z 148), and the red peak represents isotopically labeled salbutamol (m/z 249 → m/z 149). Injection of molar-equivalent concentrations of unlabeled salbutamol (1000 ng/mL) and labeled salbutamol (1039 ng/mL) into the LC-MS/MS system resulted in comparable peak areas for both analytes.

Optimization of chromatographic conditions was also critical for the effective separation and quantification of salbutamol. The column type and mobile phase were selected to achieve reasonable retention times (Rt) for both analytes. Retention time is a key parameter used alongside exact mass, isotopic pattern, and library matching for reliable compound identification. The chromatograms in Figures 3 and 4 demonstrate successful separation of unlabeled and labeled salbutamol in aqueous and urine matrices. Comparable peak areas for the molar-equivalent mixture of 1000 ng/mL unlabeled salbutamol and 1039 ng/mL labeled salbutamol support the reliability of the method for salbutamol quantification in different matrices.

**Figure 5.**
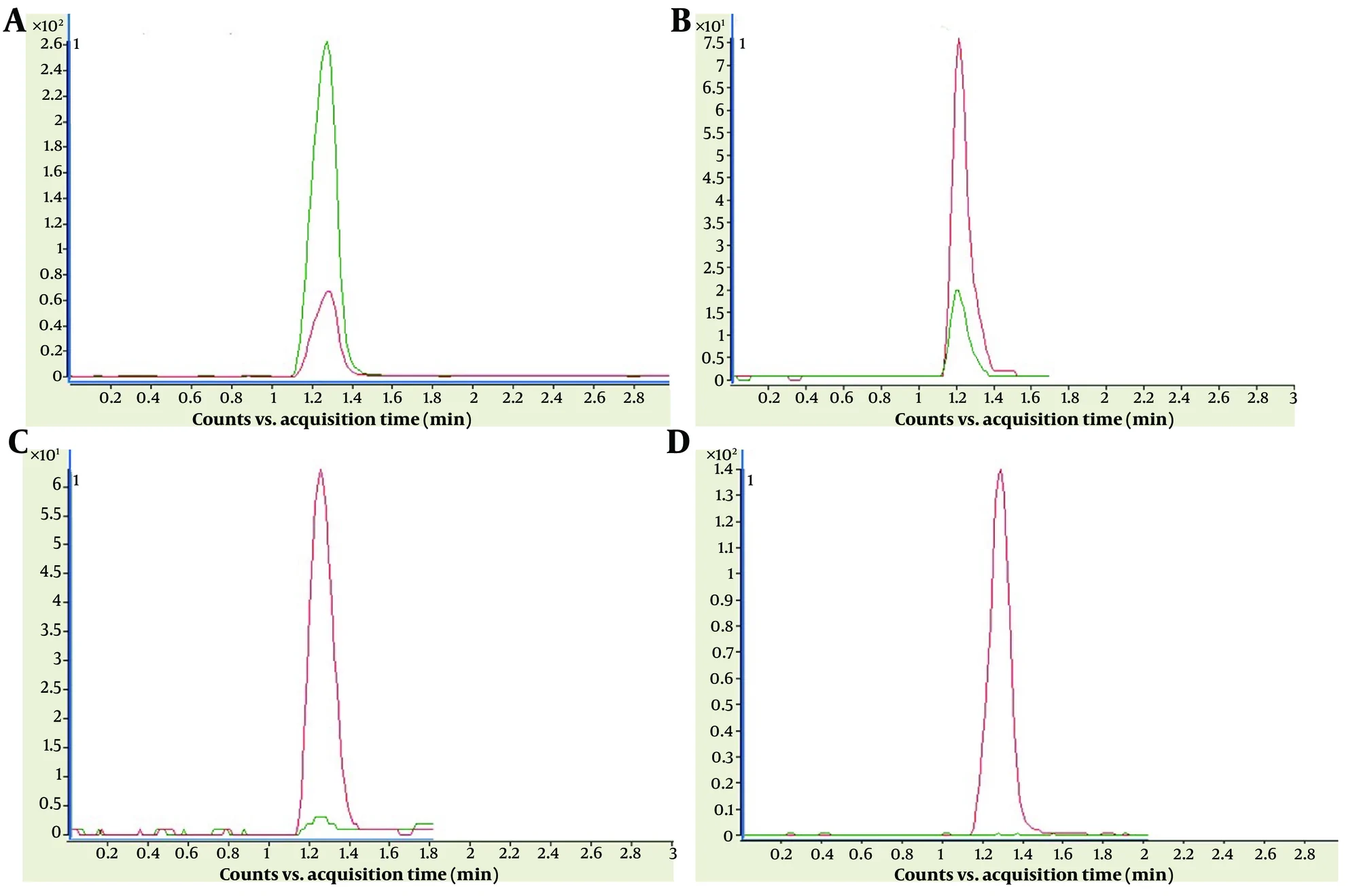
Chromatograms of human urine samples collected at different time points following salbutamol administration. The green peak represents salbutamol, and the red peak represents isotopically labeled salbutamol (salbutamol-d9). (A) 3 hours after administration; (B) 24 hours after administration; (C) 48 hours after administration; and (D) 72 hours after administration.

Analysis of human urine samples provided information on the pharmacokinetics of salbutamol. As shown in Figure 5, salbutamol was detectable in human urine up to 24 hours after administration, with a higher peak area for the unlabeled form than for the labeled form. This finding is consistent with the expected pharmacokinetic profile of salbutamol, in which the drug is rapidly absorbed and metabolized, resulting in measurable urinary concentrations over an extended period (23). The ability to detect salbutamol in human samples with high sensitivity and specificity supports the potential of this method for therapeutic drug monitoring and pharmacokinetic studies. Moreover, this streamlined approach can shorten prohibited-substance screening and reduce the number of steps by eliminating the need to repeat method development and validation for different biological samples. Despite differences in participants' diets, the results showed a consistent pattern, and the urine matrix did not interfere with the analysis.

**Figure 6.**
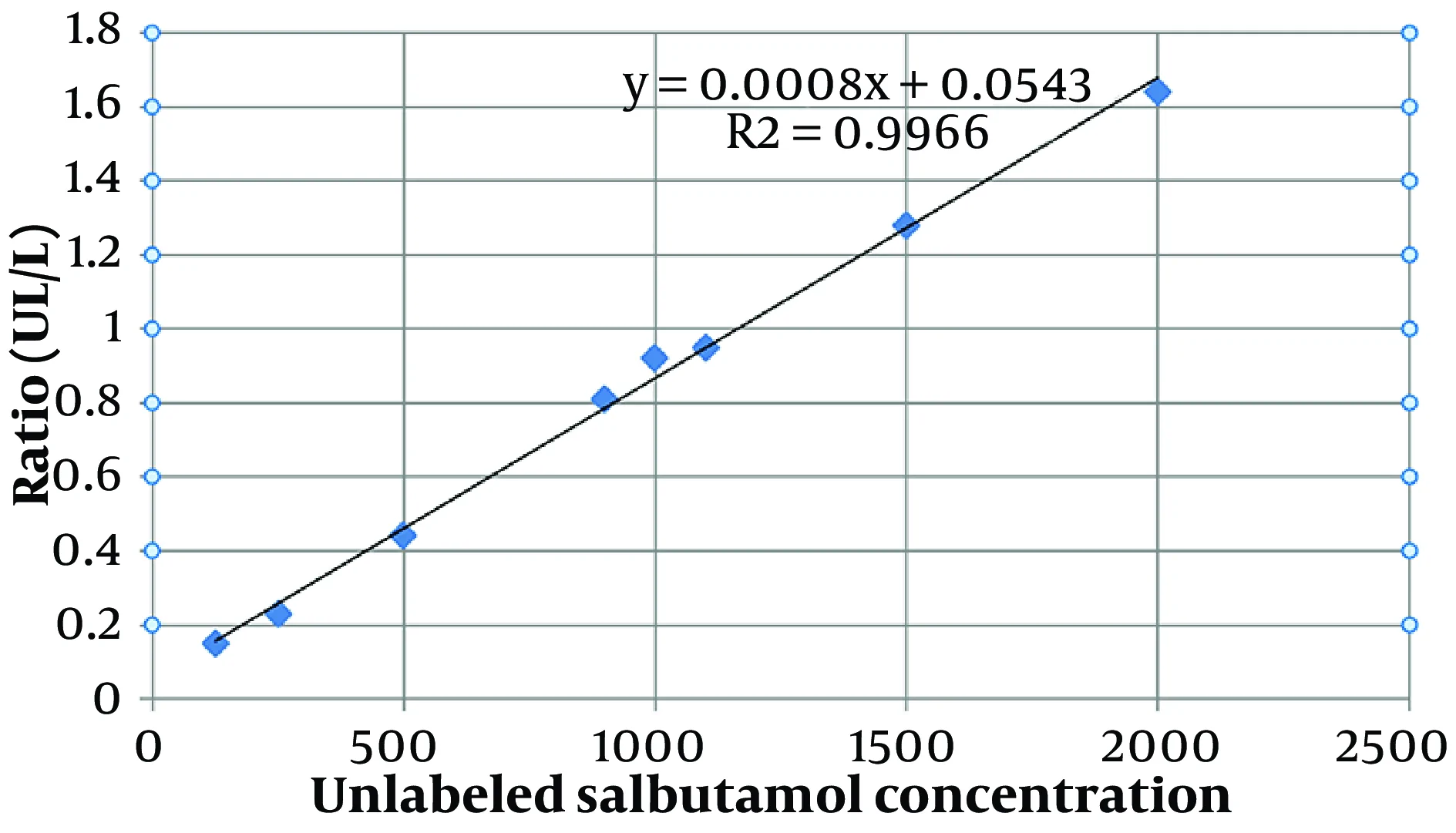
Linearity of the calibration curve. Calibration curve of unlabeled-analyte concentration versus the ratio of the unlabeled-analyte peak area (UL) to the labeled-analyte peak area (L). The linear regression equation and r^2^ value were calculated. The high correlation coefficient (R^2^) indicates a strong linear association between the concentration of unlabeled salbutamol and the corresponding peak area.

The linearity of the calibration curve, shown in Figure 6, further demonstrates the robustness of the method. A clear linear relationship was observed for salbutamol with an appropriate regression constant. The high correlation coefficient (R^2^) indicates a strong linear association between salbutamol concentration and the corresponding peak area, supporting accurate quantification across a wide concentration range. This linearity is important for applications at both low and high concentrations. These findings are consistent with previous studies reporting strong linear relationships for beta-2 agonists, including salbutamol, over broad concentration ranges. Notably, the study achieved coefficients of determination (R^2^) exceeding 0.9966 for all analytes, further underscoring the robustness of the UHPLC-Q-Orbitrap HRMS method.

The intra-day and inter-day precision analyses shown in Tables 2-5 underscore the method's reproducibility and reliability. The low relative standard deviation (RSD) values, combined with high accuracy percentages, confirm the method's consistency and precision across single-day and multi-day analyses. Achieving RSD values below 8% in intra-day and inter-day repetitions (except for one result) demonstrates the method's exceptional precision. In addition, recovery ranging from 95% to 115% (except for one result) further validates the accuracy and correctness of this method. This level of precision and accuracy is critical for obtaining reliable results in clinical and research applications.

**Table 2.** Intra-Day Analysis of Precision and Recovery Rates (Day 1) ^a^

Sample No.	Concentration (ng/mL)	Mean Unlabeled Salbutamol Peak Area	Mean Labeled Salbutamol Peak Area	SD	RSD (%)	RE (%)	Recovery (%)
**1**	125	683.33	3891.67	8.08	7.06	-8.48	108.48
**2**	250	1407.67	5468.67	3.20	1.48	-13.39	113.39
**3**	500	2750.00	6437.00	21.16	4.93	-14.20	114.20
**4**	900	4497.67	5343.00	8.12	0.86	5.22	94.78
**5**	1000	9305.67	10379.33	3.90	0.38	1.54	98.46
**6**	1100	2698.00	2766.33	8.12	0.73	1.26	98.74
**7**	1500	3122.67	2657.33	5.08	0.37	-9.09	109.09
**8**	2000	3388.00	2069.00	12.04	0.62	-2.92	102.92

^a^ Precision was evaluated by calculating RSD through repeated sample analysis. Three replicate analyses were performed for each concentration (n = 3). Accuracy was assessed by calculating RE of measured concentrations.

Coeluting compounds from the matrix or other sources can alter signal response, causing ion suppression or enhancement and resulting in poor accuracy, linearity, inter-day or intra-day precision, and sensitivity. Matrix effects and approaches for their reduction or elimination have been widely investigated in LC-MS (18, 19). Theoretically, salts and polar endogenous compounds (eg, urea, creatinine, glucuronic acid, phenol, uric acid, hippuric acid, and urobilin) in urine are responsible for substantial matrix effects (24). However, no specific matrix-effect results were reported in this study (Tables 2-5). The successful separation and quantification of salbutamol in urine samples, as shown in Figures 3-5, suggest that the optimized LC-MS/MS method effectively minimized matrix interference. Comparable peak areas for unlabeled and labeled salbutamol in urine samples (Figure 4) further support the robustness of the method in a complex matrix.

The LOD and LOQ were determined to be 0.3 ppb and 1 ppb, respectively. These low detection limits highlight the sensitivity of the method and enable the detection of salbutamol at very low concentrations. This is particularly important for applications such as doping control, where trace amounts of salbutamol must be detected in biological samples. Based on the calculated LOD and LOQ, the compounds investigated in this study can be identified and quantified at low unlabeled-analyte concentrations.

Several LC-MS/MS methods have previously been reported for the determination of salbutamol in urine, using different sample-preparation procedures, internal standards, and analytical strategies. These methods vary in sensitivity, analysis time, and their ability to compensate for matrix effects. A direct urine-injection LC-MS/MS method using an internal standard reported a lower limit of detection of 20 ng/mL and a run time of approximately 3 minutes, providing simple sample preparation but low sensitivity. A UPLC-MS/MS method using a dilution procedure achieved improved chromatographic performance with a run time of approximately 3.2 minutes and reported an LOQ of 200 ng/mL (13). Another LC-MS/MS method based on ethyl acetate extraction achieved an LOQ of 1 ng/mL with a run time of 3 - 4 minutes, showing comparable sensitivity but a longer analysis time (20). Classical LC-ESI-MS methods using solid-phase extraction and hydrolysis reported an LOQ of 10 ng/mL and analysis times of 4 - 5 minutes (21). In the present study, a surrogate analyte-based LC-MS/MS method was developed and validated for the determination of salbutamol in urine using stable isotope-labeled salbutamol-d9. The proposed method combines liquid-liquid extraction with a rapid chromatographic run time of only 2 minutes per sample and achieved an LOD of 0.3 ng/mL and an LOQ of 1 ng/mL. Compared with previously reported methods, the present approach provides superior analytical sensitivity, shorter analysis time, simplified sample preparation, and improved compensation for matrix effects through the use of a stable isotope-labeled surrogate analyte, making it suitable for routine high-throughput doping-control analysis.

**Table 3.** Intra-Day Analysis of Matrix Effects and Recovery Rates (Day 2) ^a^

Sample No.	Concentration (ng/mL)	Mean Unlabeled Salbutamol Peak Area	Mean Labeled Salbutamol Peak Area	SD	RSD (%)	RE (%)	Recovery (%)
**1**	125	530.67	4061.00	1.96	1.69	-7.56	107.56
**2**	250	1208.67	5412.33	2.10	0.96	-12.62	112.62
**3**	500	2521.33	6472.33	13.06	3.24	-19.40	119.40
**4**	900	4557.67	5722.33	11.62	1.36	-4.98	104.98
**5**	1000	9686.67	9972.67	11.42	1.09	4.95	95.05
**6**	1100	3193.33	3186.67	13.93	1.29	-1.50	101.50
**7**	1500	3107.67	2336.67	15.57	1.08	-3.46	103.46
**8**	2000	3083.33	1845.00	9.22	0.50	-8.64	108.64

^a^ Precision was evaluated by calculating RSD through repeated sample analysis. Three replicate analyses were performed for each concentration on a single day (n = 3). Accuracy was assessed by calculating RE of measured concentrations.

**Table 4.** Intra-Day Analysis of Matrix Effects and Recovery Rates (Day 3) ^a^

Sample No.	Concentration (ng/mL)	Mean Unlabeled Salbutamol Peak Area	Mean Labeled Salbutamol Peak Area	SD	RSD (%)	RE (%)	Recovery (%)
**1**	125	630.33	4156.00	12.68	10.41	-2.56	102.56
**2**	250	1254.33	5545.67	1.18	0.55	-14.06	114.06
**3**	500	2807.00	6357.00	9.77	2.02	-3.12	103.12
**4**	900	4497.67	5577.00	10.28	1.09	4.47	95.53
**5**	1000	9782.33	10686.67	9.29	0.86	7.64	92.36
**6**	1100	2934.33	3085.00	13.19	1.18	1.91	98.09
**7**	1500	3081.67	2408.33	12.36	0.81	2.10	97.90
**8**	2000	3198.67	1952.00	14.29	0.72	-1.00	101.00

^a^ Precision was evaluated by calculating RSD through repeated sample analysis. Three replicate analyses were performed for each concentration on a single day (n = 3). Accuracy was assessed by calculating RE of measured concentrations.

**Table 5.** Inter-Day Analysis of Matrix Effects and Recovery Rates ^a^

Sample No.	Concentration (ng/mL)	Mean Unlabeled Salbutamol Peak Area	Mean Labeled Salbutamol Peak Area	SD	RSD (%)	RE (%)	Recovery (%)
**1**	125	614.33	4036.00	28.16	22.98	-1.96	101.96
**2**	250	1289.67	5475.00	19.29	8.52	-9.47	109.47
**3**	500	2692.67	6422.00	27.43	6.01	-8.77	108.77
**4**	900	4517.00	5547.33	29.73	3.13	5.60	94.40
**5**	1000	9591.00	10345.67	39.70	3.64	9.16	90.84
**6**	1100	2941.67	3012.33	31.98	2.78	4.74	95.26
**7**	1500	3103.33	2467.00	98.83	6.55	0.58	99.42
**8**	2000	3223.00	1955.33	23.89	1.20	-0.35	100.35

^a^ Precision was evaluated by calculating RSD through repeated sample analysis conducted over 3 different days. Accuracy was assessed by calculating RE of measured concentrations.

## 5. Discussion

The use of LC-MS/MS-based methods in doping laboratories to analyze urine samples for salbutamol presents significant challenges, particularly matrix effects, which can lead to deviations from true values. This limitation may reduce the efficiency and accuracy of current methods used in doping-control laboratories and may potentially lead to incorrect results. To address this issue, the present study introduces a method designed to compensate for matrix effects and improve the accuracy of doping-substance analysis across different biological matrices. By mitigating the impact of matrix differences, the proposed method provides improved reliability and consistency. This method eliminates the need for traditional calibration curves, simplifies quantification, and accelerates the analytical process. The surrogate analyte screening method is particularly useful for substances with defined legal limits and available labeled isotopes. As an alternative to the official methods recommended by WADA, this approach may enable faster and more efficient analysis of prohibited substances and improve the reliability of doping-control efforts. The method’s broad applicability and precision may represent an advancement in doping analysis.

### 5.1. Limitations and Future Perspectives

Despite the satisfactory analytical performance of the proposed surrogate analyte-based LC-MS/MS method, several limitations and future perspectives should be considered. The present study included a limited number of human volunteers (n = 4); therefore, future investigations involving larger and more diverse populations are required to better evaluate interindividual variability in salbutamol excretion. In addition, the method relies on a labeled analyte, which may increase analytical costs and limit routine implementation in some laboratories. Future research may also explore the applicability of this surrogate analyte approach to other biological matrices, such as plasma, blood, or sweat. Importantly, this methodology could be extended to other doping-related substances with established legal thresholds and available labeled analytes, thereby broadening its applicability in clinical and anti-doping analysis.

### 5.2. Conclusions

The surrogate analyte-based LC-MS/MS method may improve the reliability and efficiency of salbutamol screening by compensating for matrix and sample-preparation effects and simplifying threshold-based interpretation. Its applicability to other biological matrices and other doping-related substances with established legal thresholds and available labeled isotopes warrants further investigation.

## Data Availability

The dataset presented in the study is available on request from the corresponding author during submission or after publication.
